# Innovative community-based ecosystem management for dengue and Chagas disease prevention in low and middle income countries in Latin America and the Caribbean

**DOI:** 10.1093/trstmh/tru201

**Published:** 2015-01-19

**Authors:** Jacobo Finkelman

**Affiliations:** Estado de Mexico, Mexico

**Keywords:** Chagas disease, Dengue, Latin America, Vector control

## Abstract

In 2009, the WHO Special Programme for Research and Training in Tropical Diseases (TDR) and the International Development Research Centre (IDRC) launched a call for innovative community-based ecosystem management research projects for dengue and Chagas disease prevention in low and middle income countries in Latin America and the Caribbean. Eight research institutions were selected. The outputs of these projects led to a better understanding of the interaction between ecological, biological, social and economic (eco-bio-social) determinants of dengue and Chagas disease in Latin America and the Caribbean. Both diseases are considered highly relevant in the regional health agendas.

Dengue, caused by any of the four dengue virus serotypes, is regarded as the most important arboviral disease globally. More than 50% of the world's population lives in regions at risk of the disease, and evidence points towards further geographical and numerical expansion.^1^ The Region of the Americas is not an exception; dengue incidence has increased 30 times in the last 50 years. Between 2008 and 2012, more than 1.2 million cases of dengue were notified annually, including 28 233 severe cases and 1000 deaths. Furthermore, 2013 had the highest burden of disease ever registered, with the largest epidemic in the history of the Americas, with a total of over 2.3 million cases, almost 38 000 severe cases and 1318 deaths.^2^ Nevertheless, in the Americas, the case fatality rate (CFR) due to severe dengue has declined over the past 3 years showing the lowest CFR of any WHO region (0.055%).^3^

This disease has a high social and economic impact, affecting not just the patient, but also families and the community as a whole. In the Americas, the estimated economic cost of the disease supersedes US$2.1 billion per year.^4^ An analysis of dengue cases and its social and economic determinants revealed that countries with higher levels of social inequality (Gini index), illiteracy, and populations living without access to water and sanitation services also had the highest prevalence of dengue.^3^

The Pan American Health Organization (PAHO)/WHO supports member states in the implementation of the Integrated Management Strategy for the Prevention and Control of Dengue (IMS-Dengue) by strengthening the epidemiological surveillance, laboratory networks, integrated vector management, clinical management of patients, environmental management and social communications.^3^

More recently, the WHO *Global strategy for the prevention and control of dengue 2012–2020* was launched. The goal is to reduce the burden of dengue by reducing dengue mortality by 50% and morbidity by 25% by 2020. This Global Strategy is based on five technical elements intended to work together in an integrated way: (1) diagnosis and case management, (2) integrated surveillance and outbreak preparedness, (3) sustainable vector control, (4) future vaccine implementation and (5) basic operational and implementation research; and five enabling factors for the successful implementation of the Global Strategy: (1) advocacy and resource mobilization, (2) partnership, coordination and collaboration, (3) communication to achieve behavioral outcomes, (4) capacity building and (5) monitoring and evaluation.^5^

The recurrent yellow fever outbreaks in some countries in the Americas^6^ and the emergence of Chikungunya^7^ are also pressing more than ever for cost-effective and environmentally safe vector control interventions.

There is increasing evidence that global environmental changes will have direct and indirect effects on vector borne diseases.^8^ In addition, the growth of urbanization in the tropics and increased travel and trade originating from endemic areas could also facilitate vector borne disease transmission.^9^

In view of this panorama and considering the potential difficulties of developing an effective dengue vaccine for public health use, cross-disciplinary research on the ecological, biological and social risk factors for dengue vector breeding and virus transmission continues to be important. As does the development and validation of sustainable interventions that do not exclusively rest on governmental control services but involve other actors such as local enterprises, social services, schools and action groups from different community strata making clear that dengue prevention and control is not a problem exclusive to the health sector. The findings of the dengue projects supported by the TDR/IDRC eco-bio-social initiative provide useful insights that may contribute to a more sustainable approach towards its prevention and control.

In 2010, PAHO Directing Council extended the goal for Chagas disease elimination by interrupting domestic vector borne transfusion and other types of *Trypanosoma cruzi* transmission in all subregions of the Americas by 2015.^10^ Although substantial efforts have been made, countries where Chagas disease is endemic are now actively engaged in updating their epidemiologic data in order to assess their compliance with the elimination goal. Yet, based on limited information it is very likely that the elimination target date might be subject for further review.

Chagas is a neglected disease. Its transmission is associated with multiple social and environmental determinants. Salient among the main determinants are poor-quality dwellings—chiefly in rural and suburban areas—and living in areas marked by poverty, social instability or high migration rates. The disease is also associated with populations involved in seasonal agricultural work harvesting sugarcane and other crops. This disease contributes to perpetuating the cycle of poverty, since it reduces learning, productivity and earning capacity.^11^ Between 2002 and 2012, populations living below extreme poverty and poverty levels in Latin America dropped form 19.3% and 43.9% to 11.3% and 28.2%, respectively,^12^ yet millions of people are still exposed to *T. cruzi* infection.

Because *T. cruzi* has a sylvatic cycle it will be impossible to totally eliminate *T. cruzi* infected vectors as well as in non-human reservoirs. Environmental factors, combined with the presence of triatomine vectors, makeshift dwellings and human exposure, have created the conditions for perpetuating the effective transmission of the infection and the re-infestation of areas where the vectors have been previously eliminated. Although less frequently, the infection is also transmitted through transfusions, organ donations, from mother to child through the placenta and, more rarely, by contaminated food.^11^

Lack of clinical symptoms makes Chagas a silent disease that often goes undiagnosed, with little attention paid to it in medical schools and training centers for health professionals. Although Chagas disease has been endemic in the Americas only, the migration of infected people can transport the disease to non-endemic countries of the Americas and the rest of the world. Even if transmission was interrupted today, the chronic sequelae will continue to place a large economic and social burden on Chagas endemic countries.^13^

Considering the diversity of Chagas vector behavior and the variety of environmental and social conditions of the populations in endemic or re-infested areas underline the importance of intervention research to develop locally adapted control strategies. The implementation research conducted by the TDR/IDRC supported research teams is contributing to the overall goal of eliminating Chagas disease from the Americas.
